# On the Choice of Medical Books

**Published:** 1817-03

**Authors:** 


*For the London Medical and Physical Journal.*

### *On the Choice of Medical Books*; by SENEX, (*not the former.*)

YOUR Young Friend but Constant Reader's Inquiry concerning the Choice of Medical Books, ought to receive every attention and encouragement from those brethren of the profession, whose experience may advantageously direct his studies.

I lament his complaint against his master, who, though willing to allow him the use of his library, yet withholds his advice as to the most eligible books which should occupy his vacant hours.

I will mention a few excellent elementary volumes; and, in a subsequent communication, will extend my observations on the future advancement of his studies.

I have

I have been in the habit of recommending to my pupils a complete knowledge of, and a correct mode of translating, the last editions of the London and Edinburgh Pharmacopœias; at the same time attentively reading the excellent Edinburgh and London Dispensatories of Dr. Duncan, jun. and Mr. Todd Thompson.

An useful little book, published by the late Mr. Lucas, of Leeds, entitled "The Qualifications of a Surgeon-Apothecary;" and Mr. Chamberlaine's "Tyronium Medicum," are also interesting works for a young man entering upon the study of the mixed branches of the profession.

After the first and second years of an apprenticeship, the elementary principles of anatomy, surgery, medicine, and botany, may be begun. On these subjects, the works of Fyfe, Monro, the Bells, Cullen, Abernethy, Withering, Stokes, Astley Cooper, and many others whose works I may hereafter specifically mention, may be studied with manifest advantage.

In the more leisure hours of your Young but Constant Reader, a perusal of Hutchinson's Biographia Medica will afford him many excellent and valuable examples of medical characters whose zeal and industry had elevated them to the highest stations in their professions, and of others whose works had ennobled the science, and improved the practice of each department. Aikin's Memoirs of Medicine may be consulted with equal advantage.

Your Young Friend will have sufficient employment, during the first year of his apprenticeship, by an attentive perusal of the elementary volumes I have now mentioned; and I will take an early opportunity of renewing the subject.

*Liverpool; Feb.* 9, 1817.

